# Density Gradient Selection of Colloidal Silver Nanotriangles for Assembling Dye-Particle Plasmophores

**DOI:** 10.3390/nano9060893

**Published:** 2019-06-18

**Authors:** Rui Oliveira-Silva, Mariana Sousa-Jerónimo, David Botequim, Nuno J. O. Silva, Duarte M. F. Prazeres, Pedro M. R. Paulo

**Affiliations:** 1 iBB-Institute for Bioengineering and Biosciences, Instituto Superior Técnico, Universidade de Lisboa, Av. Rovisco Pais 1, 1049-001 Lisboa, Portugal; ruipsilva@tecnico.ulisboa.pt (R.O.-S.); mariana.jeronimo@tecnico.ulisboa.pt (M.S.-J.); davidbotequim@tecnico.ulisboa.pt (D.B.); miguelprazeres@tecnico.ulisboa.pt (D.M.F.P.); 2 Departamento de Física and CICECO, Aveiro Institute of Materials, Universidade de Aveiro, 3810-193 Aveiro, Portugal; nunojoao@ua.pt; 3 Centro de Química Estrutural, Instituto Superior Técnico, Universidade de Lisboa, Av. Rovisco Pais 1, 1049-001 Lisboa, Portugal

**Keywords:** colloidal metal nanoparticles, density gradient centrifugation, plasmonics, fluorescence correlation spectroscopy, plasmophores

## Abstract

A simple method based on sucrose density gradient centrifugation is proposed here for the fractionation of colloidal silver nanotriangles. This method afforded particle fractions with surface plasmon resonances, spanning from red to infrared spectral ranges that could be used to tune optical properties for plasmonic applications. This feature was exemplified by selecting silver nanotriangle samples with spectral overlap with Atto-655 dye’s absorption and emission in order to assemble dye-particle plasmophores. The emission brightness of an individual plasmophore, as characterized by fluorescence correlation spectroscopy, is at least 1000-fold more intense than that of a single Atto-655 dye label, which renders them as promising platforms for the development of fluorescence-based nanosensors.

## 1. Introduction

The conjugation of fluorescent dyes onto metal nanoparticles is a promising approach to obtain strongly emitting nanoprobes with emission intensities far superior to their isolated components. The metal nanoparticle can act as an optical antenna through its localized surface plasmon modes, thereby modifying the dye’s emission properties by accelerating its excitation and decay rates [1,2,3,4,5]. The overall effect depends on the details of the plasmon near field close to the particle’s surface, and it may comprise more than intensity changes [6,7,8,9,10]. The role of plasmon-molecule interactions in the emergence of unique emission properties in these systems has been adequately captured in the term plasmophore [11,12,13,14,15].

Several aspects have to be considered in order to achieve substantial emission enhancements from a plasmophore. For instance, the dye-particle separation is crucial, because at very close distances, quenching by the metal surface mostly overcomes the near field enhancement effects on the dye’s excitation and radiative decay rate [1,2]. Typically, there is an optimal location for emission enhancement a few nanometers away from the metal surface. Another key aspect is the spectral dependence of plasmon-coupled emission [5,16,17]. The largest effects occur when the surface plasmon resonance of the nanoparticle is close to the dye’s excitation wavelength and, correspondingly, the dye’s emission is slightly red-shifted relatively to the plasmon peak wavelength. For a particular dye, the spectral overlap condition can be attained by changing the particle’s composition, size, or shape in order to tune its surface plasmon resonance.

The development of wet-chemistry methods for the synthesis of colloidal metal nanoparticles offers the possibility of preparing a variety of particle types and, therefore, to tune its optical properties [18,19,20]. For the purpose of fluorescence enhancement, gold and silver are the most commonly used metals. The dielectric properties of silver, namely its lower dissipation in the visible range, allow for larger plasmon enhanced fields that yield better antenna effects. However, silver nanoparticles are prone to oxidation and, thus, gold is preferred because of its chemical stability. Elongated particle shapes of gold or silver, such as rods, bipyramids, prisms, or stars, display surface plasmon resonances that span from red to infrared spectral ranges for which dissipation is weak in both metals. This results in surface plasmon resonances with strong near field enhancements that are mostly concentrated at the sharp features on the particle’s surface, which are designated as plasmon hot-spots. Therefore, besides the dye-particle separation and their spectral overlap, it is also important to selectively attach the dye at plasmon hot-spots to maximize antenna effects in the plasmophores’ emission.

The colloidal synthesis of elongated metal nanoparticles is usually a sequential process in which seed particles are first produced and then grown anisotropically or reshaped in the presence of surfactants or other surface agents [18]. This often leads to a mixture of particle sizes and shapes that needs to be purified or processed in order to yield samples displaying narrow plasmon resonances suitable for plasmonic applications [21,22,23,24,25,26]. Despite, silver triangular nanoplates being synthetized in a single step, morphological and size heterogeneity are still observed. Therefore, in this contribution, we propose a simple work-up procedure based on nanoparticle centrifugation in a density gradient of aqueous sucrose solutions. The procedure was demonstrated here for the selection of silver nanotriangles, but it could be readily adapted for separating silver nanoparticles of other shapes. The particle selection criterion was the spectral overlap of the plasmon resonance with Atto-655 dye’s absorption and emission in order to develop red-emitting plasmophores. For this purpose, silver nanotriangles were coated with a thin gold shell to improve their chemical stability and labeled with Atto-655 dye covalently attached on a DNA oligonucleotide sequence with 10-nt and terminated by a thiol group. The strong emission properties of the assembled dye-particle plasmophores were confirmed by fluorescence correlation spectroscopy measurements.

## 2. Materials and Methods

### 2.1. Reagents

Silver nitrate (AgNO_3_) was acquired from Panreac Appliechem (Ottoweg, Darmstadt, Germany), trisodium citrate (99.95%), hydrogen peroxide (H_2_O_2_, >30%) and sodium hydroxide (NaOH, 99.1%) from Fisher Scientific (Loughborough, Leicester, United Kingdom), sodium sulfite (Na_2_SO_3_, 97%) from Acros Organics (New Jersey, USA), sucrose (>99%) and gold(III) chloride solution (HAuCl_4_, >49% Au bases) from Sigma-Aldrich (St. Louis, MO, USA), polyvinylpyrrolidone (PVP, MW = 10,000) and L-(+)-ascorbic acid (L-AA, >99%) from Alfa Aesar by Thermo Fischer Scientific (Erlenbachweg, Kandel, Germany), and sodium borohydride (NaBH_4_) from Aldrich. DNA oligonucleotides of 10-nt labeled with ATTO-655 dye and purified by HPLC was purchased from STAB Vida (Monte da Caparica, Portugal). The sequence used was the following: (ATTO-655)-5′-GAGTCTGGAC-(C6-SH)-3′. Ultrapure water (18.2 MΩ⋅cm) was obtained with a Milli-Q purification system (Merck-Millipore) and used in all preparations. All reagents were used as acquired.

### 2.2. Synthesis of Silver Nanotriangles

The synthesis of colloidal silver nanotriangles has been previously reported in the literature [27]. However, the amount of H_2_O_2_ was adjusted due to aging effects on the concentration of this reagent. The reaction mixture was prepared from 100 µL of 0.1 M AgNO_3_, 1.5 mL of 0.1 M trisodium citrate, and 900 μL of H_2_O_2_ that were diluted to a final volume of 100 mL with ultrapure water. The solution was vigorously stirred for 10 min. Then, the stirring speed was decreased, and 1 mL of 0.1 M NaBH_4_ was injected. The stirring was sustained until a color variation was observed. Subsequently, the solution was centrifuged at 9000 g for 30 min. The supernatant was discarded, and 2 mL of the nanotriangle solution was retrieved.

### 2.3. Separation of Silver Nanotriangles by a Sucrose Density Gradient Centrifugation

Nanotriangles in the reaction mixture were fractionated by sucrose density gradient centrifugation. A centrifuge tube was first prepared by successive additions of 1 mL volumes of sucrose aqueous solutions starting from a concentration of 100% (w/v) at the bottom and decreasing in steps of 10% upwards. One milliliter of the reaction mixture was added at the top of the sucrose density gradient, and the separation was promoted by gentle centrifugation (6000 g, 30 min). After centrifugation, nanoparticle fractions were collected by carefully pipetting out volumes of 200 μL from the centrifuge tube along the established gradient profile.

### 2.4. Gold Coating of Silver Nanotriangles

The gold coating protocol used here was adapted from Ref. [28] with the aim of minimizing the formation of gold nanoparticles. For this procedure, selected fractions of silver nanotriangles were first combined to give three samples: Ag-1 (F2, F3, F4), Ag-2 (F6, F7, F8), and Ag-3 (F9, F10). After gold coating, these were labeled Ag@Au-1 to 3, respectively. A growth solution (4.72 mL ultrapure H_2_O, 20 μL of 0.25 M HAuCl_4_, 240 μL of 0.2 M NaOH, 3 mL of 0.01 M Na_2_SO_3_) was prepared beforehand and incubated overnight at room temperature (RT). Afterwards, 500 μL of silver nanotriangles solution were added to a solution containing 637.5 μL of ultrapure H_2_O, 250 μL of 5% (w/w) PVP, 50 μL of 0.5 M ascorbic acid, 50 μL of 0.5 M NaOH, 12.5 μL of 0.1 M Na_2_SO_3_, and 1 mL of growth solution. The mixture was kept under stirring at RT for 10 h. The resulting nanoparticles were then centrifuged at 6000 g for 30 min.

### 2.5. Functionalization with Atto-655 Labeled Oligonucleotide

The plasmophores were prepared by incubating 50 μL of gold-coated silver nanotriangles with 250 nM of the thiolated oligonucleotides labeled with Atto-655 in 10 mM of cetyltrimethylammonium bromide (CTAB) during 1 hr at RT. The resulting particles were centrifuged 3 times at 6000 g for 15 min against a solution of 1 mM CTAB.

### 2.6. Equipment

Extinction spectra were measured with an UV/Vis spectrophotometer from PerkinElmer, model Lambda 35. Corrected fluorescence emission spectra were recorded with a FluoroLog-3 spectrofluorimeter (Horiba Jobin Yvon, Tokyo, Japan). Scanning transmission electron microscopy (STEM) measurements were performed on a TEM/STEM Hitachi microscope model HD2700 operating at 200 kV, equipped with EDS and HAADF detector. Fluorescence correlation spectroscopy (FCS) measurements were performed on a time-resolved confocal fluorescence microscope (MicroTime 200, PicoQuant GmbH, Berlin, Germany). The microscope setup details were previously described [29]. Briefly, the excitation source is a pulsed diode laser emitting at 639 nm with a repetition rate set to 20 MHz. The excitation light is coupled into an inverted microscope (Olympus IX 71) through a water immersion objective (UPLSAPO 60×, N.A. 1.2, Olympus, Tokyo, Japan) and the emitted light is collected in the reverse pathway and is cleaned by an emission filter (Chroma 695AF55 with a transmission window of 668–723 nm) and a 50 μm pinhole that rejects out-of-focus light. Then, the re-collimated beam is divided by means of a 50/50 polarizing beam splitter cube and is detected by two single-photon avalanche diode detectors (SPAD, SPCM-AQR-13, Perkin Elmer, Waltham, MA, USA). The signal is processed by TimeHarp 200 TC-SPC PC-board (PicoQuant GmbH). Data acquisition and analysis were performed in SymPhoTime software, version 5.3.2.2 (PicoQuant GmbH).

### 2.7. Details of DDA Simulations

The method of discrete dipole approximation (DDA) was used to calculate the near-field enhancement in gold-coated silver nanotriangles [30]. The particle geometry used was a triangle of equal sides for the silver core covered in every surface by a gold shell with truncated tips. The triangle’s side and height were 70 and 30 nm, respectively, with a shell thickness of 2 nm. The particle volume was discretized as an array of point dipoles centered in cubic volume elements with size of 0.5 nm. The dielectric function of silver and gold used in these calculations are those reported in references [31,32], respectively. The values of dielectric function were divided by the dielectric constant of water to simulate the surrounding environment. The near-field intensity maps were calculated using the subroutine implemented in the ADDA software package [33].

## 3. Results and Discussion

**Figure 1:**
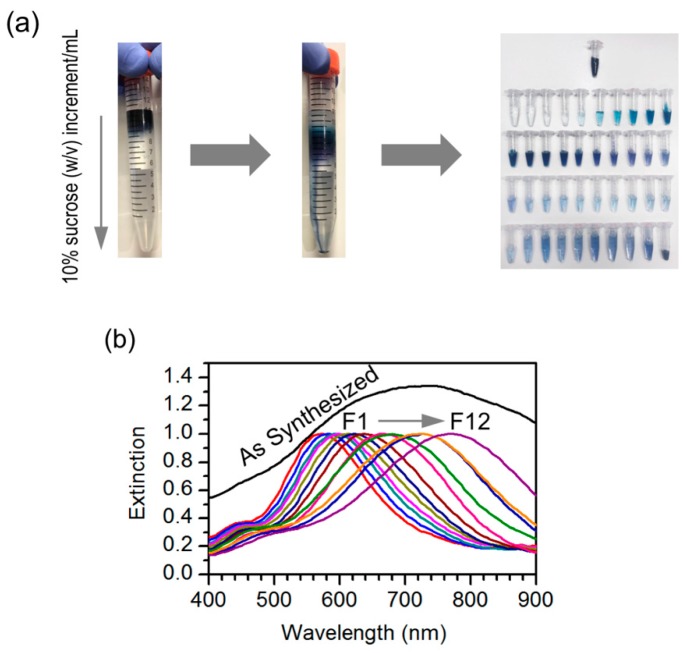
Fractionation and characterization of silver nanotriangles. (**a**) Nanotriangles were fractionated by sucrose density gradient centrifugation. The reaction mixture was placed on top of a sucrose density gradient (100–10% w/v) in a centrifuge tube; following gentle centrifugation (6000 g, 30 min), twelve 200 µL fractions were collected. Separation efficiency can be judged by the color distribution of the resulting fractions. (**b**) Normalized extinction spectrum of the original mixture and spectra of the individual fractions.

At the beginning of this work, our motivation was to evaluate the performance of silver nanotriangles as plasmonic nanoantennas for fluorescence enhancement. However, the preparation of silver nanotriangles, even if performed by a single-step well-established procedure, is very dependent on the reagents’ quality, such as hydrogen peroxide, which is unstable. This aspect can decrease reproducibility and make long-time studies difficult, for which a different synthesis has to be developed. Thus, we believe that a good separation method affording particles with consistent optical properties is important. The silver nanoparticles are easy to prepare, as indicated by the blue color that is promptly observed in the solution, but the resulting samples were too heterogeneous, as inferred from their broad extinction spectrum. With this in mind, we have decided to use density gradient centrifugation (DGC), a procedure well-established in biological sciences, to separate the resulting nanoparticles by size, which could lead to narrower optical spectra due to an improved sample homogeneity. The fractionation of the nanotriangle population synthesized was performed by adding the reaction mixture onto the top layer of a sucrose density gradient prepared in a 15 mL centrifuge tube. The centrifugation promotes particle sedimentation under the effect of opposing centrifugal and buoyancy forces. This results in the distribution of nanoparticles according to their size and shape across the density gradient. Fractions of particles of different sizes can then be obtained by collecting volumes along the centrifuge tube (Figure 1a).

Comparing the extinction spectrum of the original mixture with the spectra of separated fractions (Figure 1b), it is possible to judge that particle fractionation using DGC was successful. The original mixture displays a broad spectrum that spans a wide wavelength range from 500 to 900 nm. On the other hand, the separated fractions have much narrower spectra, and with increasing particle size, there is a gradual red-shift of the plasmon resonance peak. In the latter spectra, it also becomes possible to distinguish a smaller band or shoulder at lower wavelengths (ca. 450 nm), which is expected for the transverse plasmon mode that is excited perpendicularly to the particle’s plane. The in-plane longitudinal modes are responsible for the strong plasmon resonance peak that red-shifts with increasing particle fraction. Although an overall increase of particle size may induce a red shift in the plasmon resonance through the contribution of multipolar modes, it seems more likely that the wide range of peak shifts observed here result from an increase of the particle’s aspect ratio. This outcome from particle selection by DGC provides a way to tune the surface plasmon resonance to match different dyes across the red to infrared range when developing plasmophores.

**Figure 2:**
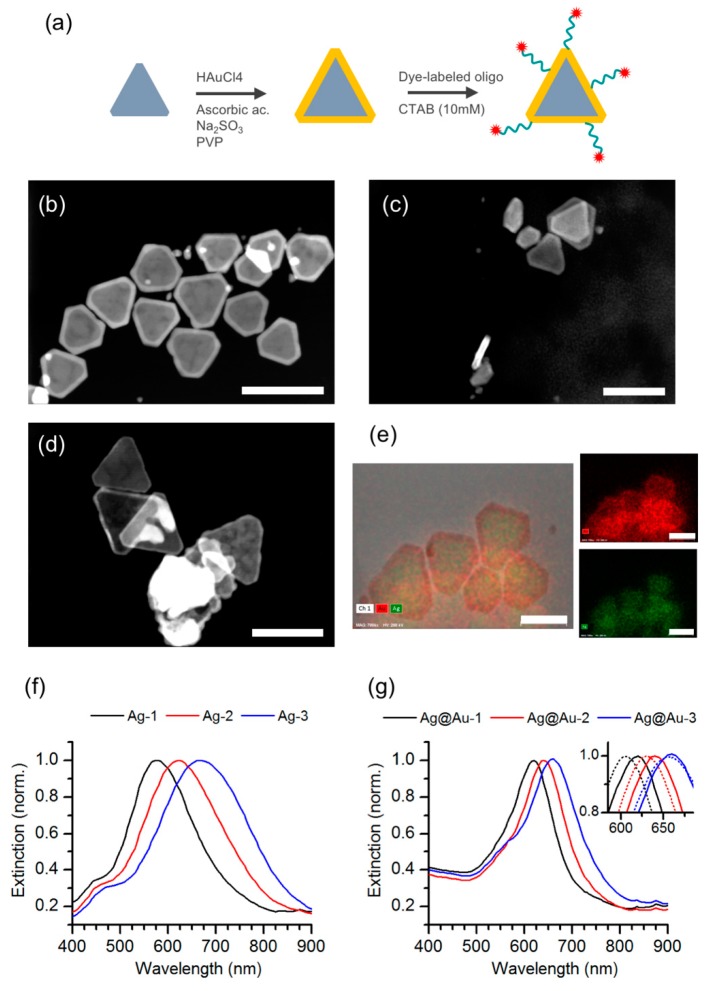
Gold coating of silver nanotriangles. (**a**) Schematic representation of gold coating of silver nanotriangles and subsequent assembly of dye-particle plasmophores; (**b**–**d**) HAADF images measured in high-angle annular dark-field imaging mode of gold-coated silver nanotriangles in samples Ag@Au-S1 to S3 (see also Supplementary Materials); (**e**) TEM and HAADF images showing elemental analysis mapping obtained by EDX of the gold-coated silver nanotriangles in sample Ag@Au-S1 (green and red represents silver and gold, respectively). The scale bar corresponds to 80 nm for all images shown here. (**f**) Normalized extinction spectra of silver nanotriangle samples Ag-1 to 3; (**g**) normalized extinction spectra of gold-coated silver nanotriangle samples Ag@Au-1 to 3. The inset shows the peak shift of the longitudinal surface plasmon upon functionalization with dye-labeled oligonucleotides (dashed lines).

Following fractionation, we selected and pooled particle fractions of silver nanotriangles with plasmon resonance peaks at around 550 nm (Ag-1), 620 nm (Ag-2), and 670 nm (Ag-3) that afford a good degree of spectral overlap with the absorption and emission from the Atto-655 dye. The silver nanotriangles in these pooled fractions (Ag-1 to 3) were then coated with a thin gold shell to improve their chemical stability (Figure 2a), yielding fractions Ag@Au-1 to 3. Indeed, the gold coating rendered these particles resistant to oxidation by hydrogen peroxide (Figure S1 of Supplementary Materials). The morphology of the gold-coated particles was characterized by STEM measurements (Figure 2b–d). After gold coating, these images show nanoprisms of triangular shape with truncated tips. The presence of an outer layer of gold surrounding the silver particle core was confirmed by HAADF images and elemental mapping using EDX analysis (Figure 2e). The selection of particle fractions by DGC was also correlated with an increase of the particle size along the density gradient (Table S1 of Supplementary Materials). The extinction spectra of samples Ag-1 to 3 also changed slightly upon gold coating by becoming narrower and with closer peak wavelengths (Figure 2f,g). The growth of a gold shell on silver nanotriangles commonly induces a red-shift of the longitudinal plasmon [28,34], but the spectral changes observed here may have also resulted from additional washing steps by centrifugation. The spectral narrowing observed in the gold-coated particles is nonetheless a beneficial feature for plasmonic applications.

After successfully obtaining Ag@Au nanoparticles, we tried to evaluate their ability to integrate a plasmophore. The assembly of dye-particle plasmophores from the selected silver nanotriangles coated with gold (Ag@Au-1 to 3) was accomplished by conjugation of Atto-655 dye-labeled oligonucleotides onto these particles. The oligonucleotide sequence is modified at the 3′-end with a thiol group to bind the metal surface, while the Atto-655 dye is attached at the 5′-end. The estimated end-to-end distance of the oligonucleotide spacer is approximately 4.3 nm, but the actual distance is smaller due to the conformational freedom of the single strand [29]. The functionalization was performed under an excess of dye-labeled oligonucleotides and in the presence of CTAB surfactant. The use of CTAB surfactant is a well-established strategy to promote tip-selective functionalization of gold nanorods [35]. This tip-preference in the presence of CTAB is attributed to a protective role of the surfactant bilayer on the rods’ surface that directs thiol attachment to more sparse regions at the tips. Some evidence that a similar effect of edge-selective thiol attachment could take place for other particle shapes has been reported in the literature [36,37]. For the dye-particle assembly sought in this work, it would be clearly beneficial to attach the dye-labeled oligonucleotides preferentially at the apex regions of the Ag@Au nanotriangles, which are hot-spots for plasmon-enhanced fluorescence emission. The extinction spectra of Ag@Au-1 to 3 samples after functionalization with the dye-labeled oligonucleotides display a slight blue shift of the longitudinal surface plasmon band (inset of Figure 2g). The partial replacement of CTAB molecules with oligonucleotide strands upon functionalization of the Ag@Au particles may have induced a change in the local refraction index, which would explain the plasmon peak shift, but we cannot exclude additional effects on the particle’s size distribution from washing by centrifugation.

**Figure 3:**
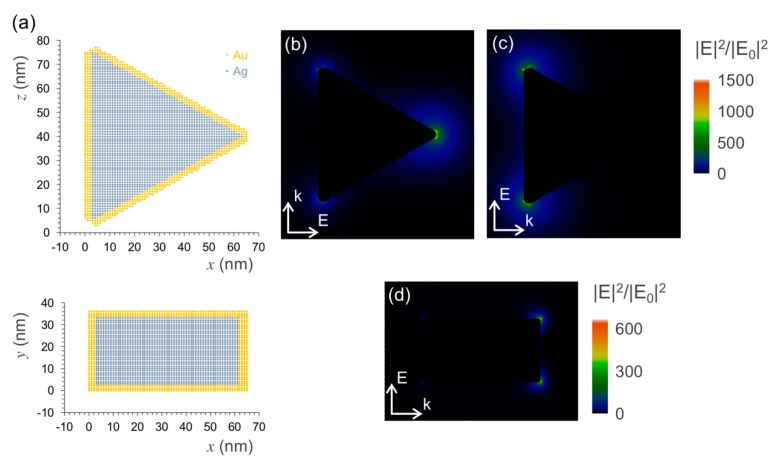
Plasmon hot-spots on the Ag@Au nanotriangles as obtained by model simulations using discrete dipole approximation. (**a**) Model of the Ag@Au nanotriangles showing particle discretization used in the simulations: (top) longitudinal and (bottom) transverse planes; (**b**) and (**c**) plasmon near field map excited at a wavelength of 639 nm by an incident field polarized in one longitudinal direction. (**d**) Similar map for an incident field polarized in transverse direction.

An approximated view of the plasmon hot-spots on the Ag@Au nanotriangles was obtained from model simulations using discrete dipole approximation. The geometry described in the literature for these particles is that of a triangular shape with truncated apexes, which is claimed to provide six hot-spots at each one of its sharp edges (Figure 3a) [28,38]. The calculated near field maps show that the distribution of the plasmon field is indeed concentrated at the triangle’s apexes for the in-plane longitudinal modes (Figure 3b,c). For an excitation wavelength of 639 nm used in the FCS measurements, the near field enhancement factors are above 1000-fold in the close proximity of the apexes aligned with the incident field direction. Moreover, the plasmon field extends with enhancement factors of at least 100-fold over a circular region that comprises a significant part of the triangle’s side edges. In the perpendicular direction to the triangle’s plane, the plasmon field at the apexes gets up to factors of 600-fold enhancement, even if the transverse mode is far from resonance at this excitation wavelength (Figure 3d). The near field enhancement factors obtained in these simulations are promising for the purpose of plasmon-enhanced fluorescence emission. These factors can be used to estimate the acceleration of the dye’s excitation rate on the particle’s vicinity. A full theoretical analysis would imply also simulating the particle’s effect on the radiative and non-radiative decay rates in order to estimate the dye-particle emission quantum yield. For a bright dye such as Atto-655, most likely it will not have much implication in the upper limit of the enhancement effect, but it could be important to map surface regions where quenching prevails. It is for this reason that site-selective approaches for dye attachment at the plasmon hot-spots are important for maximizing emission enhancement toward strongly emitting plasmophores.

**Figure 4:**
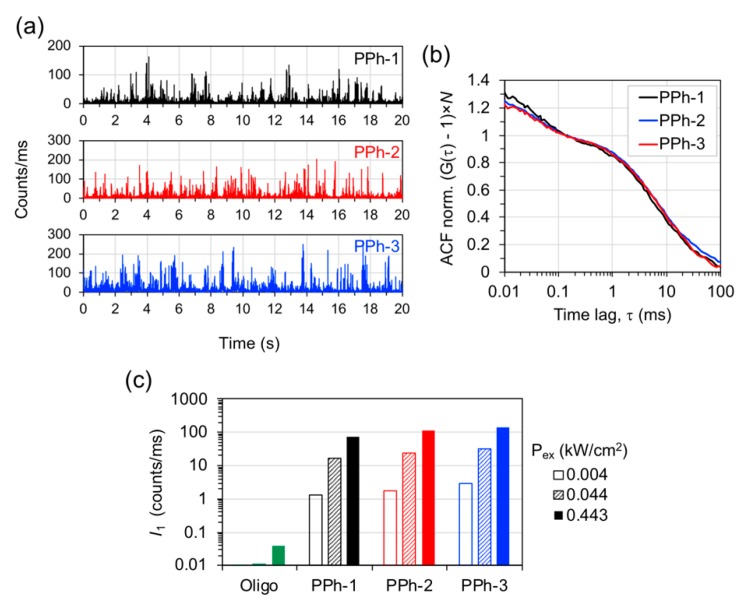
Fluorescence emission from plasmophores. (**a**) Emission intensity time traces of plasmophores PPh-1 to 3 measured in colloidal suspension for excitation at 639 nm with a power of 0.044 kW/cm^2^; (**b**) auto-correlation function (ACF) from the previous intensity time traces rescaled by the number N of emitters in the detection volume; (**c**) individual brightness ($I_{1}$) of single dye-labeled oligonucleotide (labeled “Oligo”) and of plasmophores PPh-1 to 3 obtained for several excitation powers.

The emission properties of the nanotriangle samples Ag@Au-1 to 3 conjugated to Atto-655 labeled oligonucleotides, hereafter termed plasmophores PPh-1 to 3, were characterized by FCS measurements. The intensity time traces were measured in dilute suspensions for single-particle detection and show intense fluorescence burst events that reach up to hundreds of counts/ms for a low excitation power of 0.044 kW/cm^2^ (Figure 4a). Under the same conditions, the emission from the original (non-functionalized) Ag@Au nanotriangles, as well as the emission from single Atto-655 dye molecules, is negligible. The conjugation of Atto-655 dye onto Ag@Au nanotriangles resulted in nanohybrid assemblies with single object brightness much larger than their individual components. The auto-correlation function (ACF) was obtained from each intensity trace of PPh-1 to 3 (Figure 4b). The ACF curves show two relaxation components with characteristic times of ca. 40–50 μs and 7–8 ms that were attributed to the rotational and translational Brownian motion of the dye-particle across the confocal volume. A similar behavior of the FCS correlation curves has been previously reported for the emission of dimeric gold nanoparticles conjugated to Atto-647N dye [39]. The ACF’s from PPh-1 to 3 were fitted with a two-component model for describing rotational and translational diffusion of the dye-particle assemblies:

(1) $$G\left(\tau \right)-1=\left(1-A_{s}e^{-\tau /t_{s}}\right)\times \frac{1}{N}{\left(1+\frac{\tau }{t_{d}}\right)}^{-1}{\left(1+\frac{\tau }{\kappa^{2}\cdot t_{d}}\right)}^{-1/2}$$

in which τ is the lag time, $A_{s}$ is the amplitude of the rotational correlation, $t_{s}$ is the rotational diffusion time, N is the number of emitters in the detection volume, $t_{d}$ is the transverse translational diffusion time, and κ is the ratio between the longitudinal and transverse dimensions of the detection volume. The size parameters of the detection volume were calibrated using free Atto-655 in aqueous solution as a reference dye. Using these parameters, the translational diffusion coefficients determined for PPh-1 to 3 were 8.3, 7.6, and 7.0 μm^2^/s, which, from the Stokes–Einstein equation, gave approximate values of hydrodynamic radius of 28, 30, and 33 nm, respectively. The trend observed in these results is in qualitative agreement with the density gradient selection of silver nanotriangles used for assembling PPh-1 to 3 plasmophores. However, the differences observed in diffusion coefficients are close to the experimental uncertainty typically associated with FCS measurements.

The average number N of emitters determined from ACF fittings were 0.39, 0.49, and 0.47 for PPh-1 to 3, respectively. The curves shown in Figure 4b were rescaled by these factors in order to visually compare the correlation decays of PPh-1 to 3. Furthermore, the individual brightness ($I_{1}$) of each dye-particle assembly was estimated by dividing the average emission intensity of the respective time trace by the number N of emitters (Figure 4c). The values of $I_{1}$ obtained for three excitation powers show a proportional increase from approximately 1 to 100 count/ms for the average emission of an individual object. The brightness of a single dye-labeled oligonucleotide was evaluated to be 0.039 count/ms for an excitation power of 0.443 kW/cm^2^. These results correspond to ratios of 1900-, 2870-, and 3400-fold in the individual brightness of PPh-1 to 3 relative to that of the single dye-labeled oligonucleotide. The remarkable brightness of PPh-1 to 3 is most likely due to the ability of Ag@Au nanotriangles to carry multiple dye-labeled oligonucleotides on its nanometric sized surface combined with the antenna effect on the dye’s emission at the hot-spots of these plasmonic particles. In multi-chromophore systems, the contributions of local dye concentration and that of plasmon-coupled emission are strongly convoluted in the average brightness measured. The spatial distribution of plasmon-coupled emission over the particle’s surface creates a very heterogeneous picture where strong fluorescence enhancement at the plasmon hot-spots is counter-balanced by emission quenching on other surface regions [40,41]. We have compared the emission from plasmophore PPh-3 measured in steady-state with that of the same sample after its dye molecules have been displaced from the particles’ surface into solution by ligand replacement with 2-mercaptoethanol and separated by centrifugation [42]. This comparison is based on the average emission from the ensemble of dye molecules and, thus, it is not biased by crowding of dye molecules on the Ag@Au nanoparticles, as it happens in the comparison of the emission brightness of a single particle versus that of a single dye molecule (Figure S3 of Supplementary Materials). The results show that, on average, the fluorescence emission of plasmophore PPh-3 is about three-fold more intense than that of its displaced dye, which supports the postulation that Ag@Au nanoparticles have indeed an enhancement effect on Atto-655′s emission in the dye-particle plasmophores. A detailed analysis on the spatial distribution of plasmon-coupled fluorescence is beyond the scope of this work, but recent studies using super-resolution fluorescence microscopy have contributed with valuable insights in this regard [43,44,45,46,47]. Further studies on the plasmophores demonstrated here will focus on tuning the gold nanoshell thickness and the dye labeling density. It would also be interesting to study Ag@Au nanotriangles with longitudinal plasmon resonances mismatched relatively to the dye’s optical spectrum for decoupling the effects of plasmon-enhanced emission and local dye concentration.

## 4. Conclusions

In this work, we have demonstrated a simple method for the size selection of silver nanotriangles using density gradient centrifugation (DGC) in aqueous sucrose solutions. This method made it possible to resolve a nanotriangle mixture characterized by broad extinction spectrum into twelve particle fractions displaying spectra with narrow plasmon resonances that span the red to infrared wavelength ranges. This very selective separation provides a way to tune the surface plasmon resonance for further applications. We have exemplified this possibility by selecting silver nanotriangles with plasmon resonances that overlap with Atto-655 dye’s absorption and emission in order to produce dye-particle plasmophores. The emission from these plasmophores, as characterized by FCS measurements, is higher by more than 1000-fold when compared to the emission from a single dye-labeled oligonucleotide. The remarkable brightness of the assembled plasmophores renders these as promising platforms for the development of fluorescence-based sensing schemes, in view of the large fluorescence signals that can be achieved from a single nano-object.

## Supplementary Materials

The following are available online at https://www.mdpi.com/2079-4991/9/6/893/s1. Figure S1: Resistance test of Ag@Au nanotriangles to oxidation by hydrogen peroxide. Figure S2: Extinction spectra and additional STEM images of Ag@Au nanotriangles. Table S1: Geometrical parameters of Ag@Au nanotriangles measured from STEM images. Figure S3: Emission spectra of plasmophore PPh-3 and of its displaced dye-labeled oligonucleotides.
