# Supplementary material for: Density Gradient Selection of Colloidal Silver Nanotriangles for Assembling Dye-Particle Plasmophores
Source: Nanomaterials (Basel). 2019 Jun 18;9(6):893. doi: 10.3390/nano9060893 (PMC6631754; doi:10.3390/nano9060893)
Supplement: Supplementary file 1 [file nanomaterials-09-00893-s001.pdf]

Supplementary Materials

Density Gradient Selection of Colloidal Silver Nanotriangles for Assembling Dye-Particle Plasmophores

Rui Oliveira-Silva [1,2], Mariana Sousa-Jerónimo [1], David Botequim [1,3], Nuno J. O. Silva [2], Duarte M. F. Prazeres [1] and Pedro M. R. Paulo [3,*]

1 iBB-Institute for Bioengineering and Biosciences, Instituto Superior Técnico, Universidade de Lisboa, Av. Rovisco Pais 1, 1049-001 Lisboa, Portugal; ruipsilva@tecnico.ulisboa.pt (R.O.-S.); mariana.jeronimo@tecnico.ulisboa.pt (M.S.-J.); davidbotequim@tecnico.ulisboa.pt (D.B.); miguelprazeres@tecnico.ulisboa.pt (D.M.F.P.)

2 Departamento de Física and CICECO, Aveiro Institute of Materials, Universidade de Aveiro, 3810-193 Aveiro, Portugal; nunojoao@ua.pt

3 Centro de Química Estrutural, Instituto Superior Técnico, Universidade de Lisboa, Av. Rovisco Pais 1, 1049-001 Lisboa, Portugal

* Correspondence: pedro.m.paulo@tecnico.ulisboa.pt

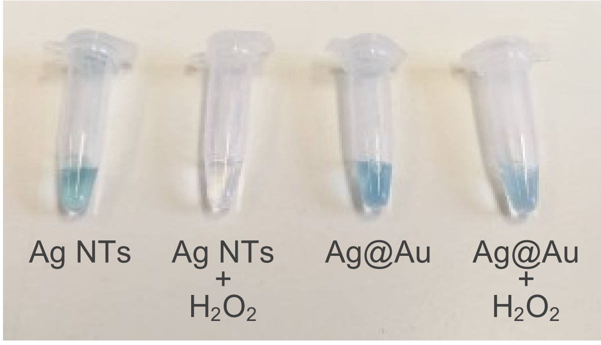

Figure S1. Confirmation of gold coating on Ag@Au nanotriangles by testing their resistance to oxidation by hydrogen peroxide. The original silver nanotriangles ("Ag NTs") when exposed to hydrogen peroxide are oxidized, which is indicated by the color loss ("Ag NTs + H_2O_2"). On the other hand, the gold coated Ag@Au nanotriangles ("Ag@Au") are resistant to oxidation ("Ag@Au + H_2O_2").

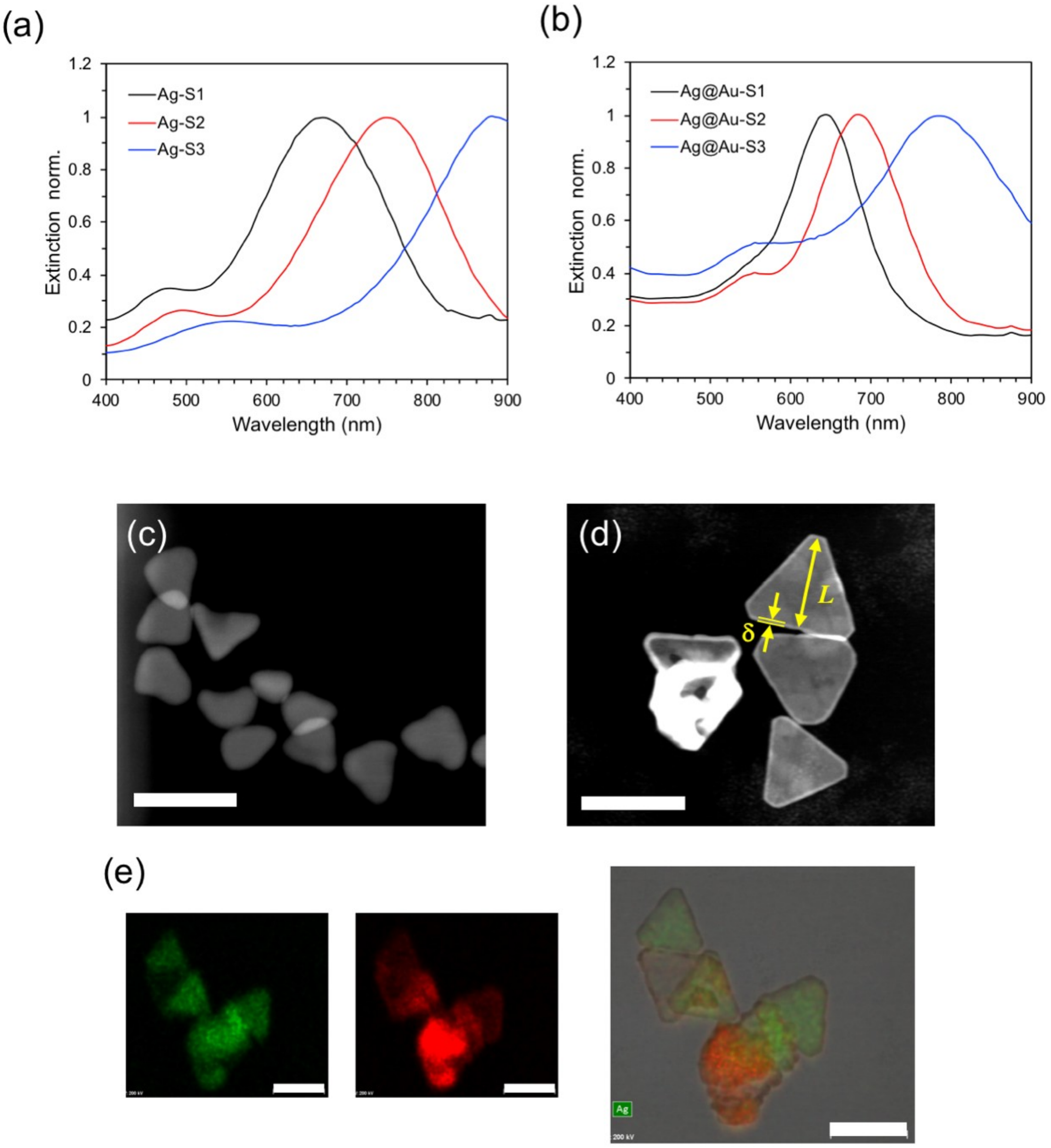

Figure S2. Normalized extinction spectra of: (**a**) silver nanotriangle samples Ag-S1 to S3, and (**b**) gold-coated silver nanotriangles prepared from the same samples Ag@Au-S1 to S3. HAADF images of (**c**) silver nanotriangle sample Ag-S3 and (**d**) of the same sample after gold coating, i.e. sample Ag@Au-S3. The yellow arrows in part (**d**) illustrate the height of the silver core measured along the triangle bisector (*L*) and the thickness of the gold coating layer (δ), that are reported below in Table S1. (**e**) TEM and HAADF images showing elemental mapping obtained by EDX analysis of the gold-coated silver nanotriangles in sample Ag@Au-S3 (green and red represents silver and gold, respectively). The scale bar corresponds to 80 nm for all images shown here.

Table S1. Geometrical parameters of gold-coated silver nanotriangles measured from STEM images of samples Ag@Au-S1 to S3. The height of the silver core measured along the triangle bisector (*L*) and the thickness of the gold coating layer (δ) were measured as illustrated in figure S2(**d**). The uncertainty interval corresponds to the standard deviation of the sample.

	L (nm)	δ (nm)
Ag@Au-S1	36 ± 5	5.1 ± 0.7
Ag@Au-S2	42 ± 6	4.6 ± 0.7
Ag@Au-S3	66 ± 9	3.1 ± 0.5

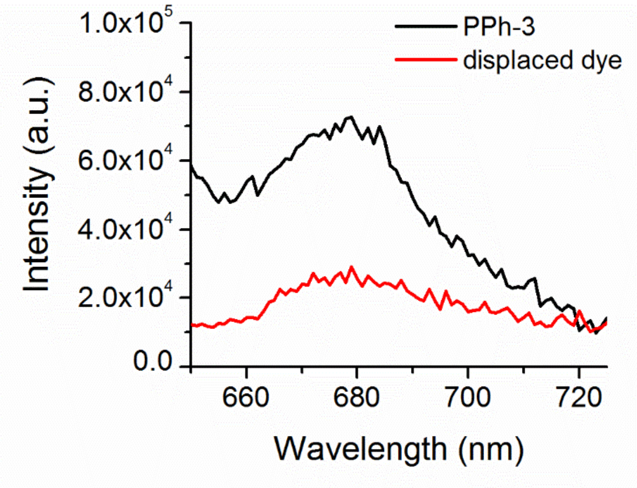

Figure S3. Emission spectrum of plasmophore PPh-3 measured in dilute suspension for excitation at 620 nm (black line) and emission of the same sample after displacing the dye-labeled oligonucleotides from the particles' surface into solution by using 2-mercaptoethanol and separating the particles by centrifugation (red line). The comparison between the emission of dye-particle nano-assemblies and that of the dye displaced in solution affords an average enhancement factor of about 3-fold for excitation of PPh-3 at 620 nm.
